# Analysis of the Expression Patterns of Tumor Necrosis Factor Alpha Signaling Pathways and Regulatory MicroRNAs in Astrocytic Tumors

**DOI:** 10.3390/ijms26125892

**Published:** 2025-06-19

**Authors:** Klaudia Skóra, Damian Strojny, Dawid Sobański, Rafał Staszkiewicz, Paweł Gogol, Mateusz Miller, Beniamin Oskar Grabarek

**Affiliations:** 1 Department of Neurological Rehabilitation, District Hospital of St. Padre Pio in Sędziszów Małopolski, 39-120 Sędziszów Małopolski, Poland; 2 Collegium Medicum, WSB University, 41-300 Dąbrowa Górnicza, Poland; drstrojny.ds@gmail.com (D.S.); drdsobanski@gmail.com (D.S.); rafalstaszkiewicz830@gmail.com (R.S.); drpawelgogol@gmail.com (P.G.); bgrabarek7@gmail.com (B.O.G.); 3 Department of Neurology, New Medical Techniques Specjalist Hospital of St. Family in Rudna Mała, 36-060 Rzeszow, Poland; 4 Institute of Health Care, National Academy of Applied Sciences in Przemyśl, 37-700 Przemyśl, Poland; 5 Department of Neurosurgery, St. Raphael Hospital, 30-693 Krakow, Poland; 6 Department of Neurosurgery, 5th Military Clinical Hospital with the SP ZOZ Polyclinic in Krakow, 30-901 Krakow, Poland; 7 Department of Neurosurgery, Faculty of Medicine in Zabrze, Academy of Silesia in Katowice, 40-555 Katowice, Poland; 8 Department of Anesthesiology and Intensive Care, Our Lady of Perpetual Help Hospital in Wołomin, 05-200 Wołomin, Poland; 9 Department of Trauma and Orthopedic Surgery, Our Lady of Perpetual Help Hospital in Wołomin, 05-200 Wołomin, Poland; 10 Pain Treatment Clinic, Our Lady of Perpetual Help Hospital in Wołomin, 05-200 Wołomin, Poland; 11 Department of Neurology, Independent Public Healthcare Institution of the Ministry of Internal Affairs and Administration in Rzeszów, 35-111 Rzeszów, Poland; mateusz.d.miller@gmail.com

**Keywords:** tumor necrosis factor alpha, astrocytoma, microRNA, MAP3K8, IL-1β, methylation

## Abstract

Chronic inflammation is increasingly recognized as a driver of glioma progression, with tumor necrosis factor-alpha (TNF-α) playing a central role in modulating the tumor microenvironment. This study aimed to investigate the expression profiles and regulatory mechanisms of TNF-α and its downstream mediators—including interleukin-1 beta (IL-1β), Mitogen-Activated Protein Kinase Kinase Kinase 8 (MAP3K8), and Mitogen-activated protein kinase kinase *7* (MAP2K7)—in astrocytic tumors of varying malignancy. We conducted an integrative molecular analysis of 60 human astrocytic tumor samples (20 G2, 12 G3, 28 G4) using transcriptomic microarrays, Reverse Transcription Quantitative Polymerase Chain Reaction (RT-qPCR), Enzyme-Linked Immunosorbent Assay (ELISA), Western blotting, immunohistochemistry, methylation-specific PCR, and miRNA profiling. Prognostic associations were evaluated using Kaplan–Meier survival and Cox regression analyses. TNF-α, IL-1β, and MAP3K8 were significantly upregulated in high-grade tumors, with log_2_ fold changes ranging from 5.56 to 8.76 (*p* < 0.001). High expression of TNF-α (HR = 2.10, 95% CI: 1.27–3.46, *p* = 0.004), IL-1β (HR = 2.35, 95% CI: 1.45–3.82, *p* = 0.001), and MAP3K8 (Hazard Ratio; HR = 1.88, 95% confidence interval; 95% CI: 1.12–3.16, *p* = 0.015) was associated with poorer overall survival. miR-34a-3p and miR-30 family members, predicted to target TNF-α and IL-1β, were markedly downregulated in G3/G4 tumors (e.g., miR-30e-3p fold change: –3.78, *p* < 0.01). Promoter hypomethylation was observed in G3/G4 tumors, supporting epigenetic activation. Our findings establish a multi-layered regulatory mechanism of TNF-α signaling in astrocytic tumors. These data highlight the TNF-α/IL-1β/MAP3K8 axis as a critical driver of glioma aggressiveness and a potential therapeutic target.

## 1. Introduction

Inflammatory signaling pathways are increasingly recognized as key contributors to the pathogenesis and progression of astrocytic tumors [1,2]. Among the most prominent molecular drivers of glioma-associated inflammation is tumor necrosis factor-alpha (TNF-α) [3], a pleiotropic cytokine that modulates various cellular processes, including proliferation, survival, differentiation, and immune responses. TNF-α primarily exerts its biological effects through its interaction with two surface receptors—TNFR1 and TNFR2—activating downstream signaling cascades such as nuclear factor kappa-light-chain-enhancer of activated B cells (NF-κB), mitogen-activated protein kinase (MAPK), and c-Jun N-terminal kinase (JNK), which are often aberrantly regulated in high-grade gliomas [4,5,6,7].

Notably, TNF-α operates at the intersection of inflammation and tumor biology. While it can promote immune-mediated tumor clearance under certain conditions, sustained TNF-α activity is frequently associated with enhanced tumor cell migration, angiogenesis, resistance to apoptosis, and maintenance of glioma stem-like cells [8,9].

Closely associated with TNF-α signaling is interleukin-1 beta (IL-1β), another critical pro-inflammatory cytokine involved in shaping the glioma microenvironment. IL-1β promotes the expression of adhesion molecules, cytokines, and growth factors that support tumor expansion and infiltration into adjacent brain tissue. It also synergizes with TNF-α in activating NF-κB-mediated transcription of inflammatory genes, thereby reinforcing the tumor-promoting inflammatory milieu [10,11,12,13].

A growing body of evidence highlights the regulatory role of microRNAs (miRNAs) in modulating TNF-α and IL-1β signaling in astrocytic tumors. These small non-coding RNAs function as post-transcriptional regulators of gene expression and play critical roles in glioma development, progression, and immune evasion [14,15]. Several miRNAs have been shown to directly target transcripts encoding TNF-α, TNF receptors, and associated signaling mediators [16,17,18], suggesting that miRNA dysregulation may significantly influence inflammatory signaling and tumor dynamics.

The epigenetic landscape further contributes to the complexity of astrocytic tumor biology. DNA methylation profiling has emerged as a valuable adjunct to histopathological evaluation, offering improved accuracy in tumor classification and prognosis. The integration of molecular and epigenetic data with traditional diagnostic criteria has refined our understanding of glioma behavior. In certain cases, tumors lacking overt histological features of malignancy may be upgraded based on molecular abnormalities alone, underscoring the growing importance of comprehensive molecular diagnostics in neuro-oncology [19].

Astrocytic tumors, which originate from astrocytes—star-shaped glial cells—are among the most prevalent primary brain neoplasms. These tumors have traditionally been classified into four histopathological grades reflecting their biological aggressiveness and clinical outcomes [20,21,22]. Despite therapeutic advancements, prognosis remains poor. Patients with lower-grade gliomas (LGGs; WHO grades II and III) generally exhibit median survival of 5 to 10 years, whereas those with high-grade gliomas, particularly grade IV, face a substantially worse prognosis, with median survival often limited to 12–24 months [23]. Glioblastoma multiforme (GBM), the most aggressive and frequent grade IV astrocytic tumor, is characterized by rapid progression and resistance to standard therapies [24,25].

In recent years, a major paradigm shift has occurred in the classification of brain tumors, driven by the integration of molecular and genetic biomarkers. The 2016 WHO classification introduced molecularly informed diagnostic criteria for central nervous system tumors, which was further refined in the 2021 update. The latter emphasized genomic features over histology, distinguishing between molecularly defined grade IV astrocytomas and glioblastomas—entities previously considered indistinguishable [26]. This approach has led to the reclassification of some tumors traditionally diagnosed as grade III based on the presence of specific genetic alterations, reflecting a more biologically meaningful stratification [27,28,29].

Prognostic evaluation and treatment decision-making in astrocytic tumors are increasingly driven by molecular biomarkers [30]. Key biomarkers include mutations in isocitrate dehydrogenase genes (IDH1/IDH2), methylation of the O6-methylguanine-DNA methyltransferase (MGMT) promoter, co-deletion of chromosomal arms 1p/19q, and amplification of the epidermal growth factor receptor (EGFR). IDH mutations, in particular, serve as powerful prognostic indicators, conferring significantly improved survival across glioma grades. For example, median survival among patients with IDH-mutant grade IV astrocytomas can reach approximately 31 months, compared to only 15 months in those with IDH wild-type tumors [31,32,33].

Therefore, the aim of this study was to evaluate variations in the expression patterns of TNF-α-related inflammatory mediators and their regulatory microRNAs in astrocytic tumors of varying malignancy grades.

## 2. Results

### 2.1. Transcriptomic Profiling of TNF-α Pathway Genes

**Figure 1:**
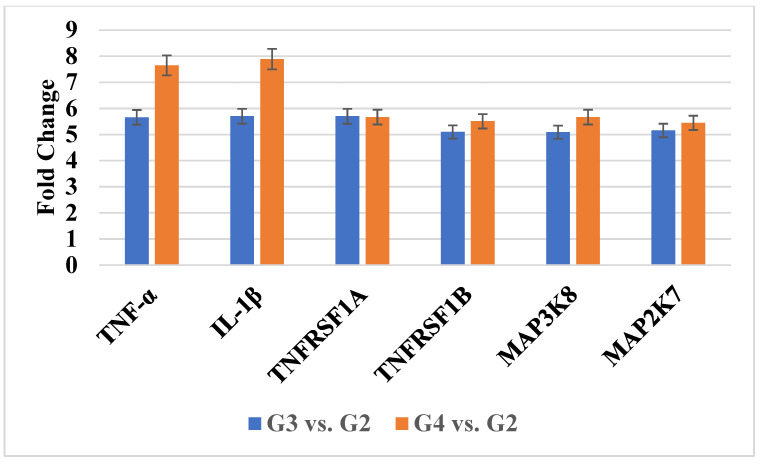
Expression profile of 6 mRNAs related to the TNF-α signaling pathways obtained via RTqPCR. TNF-α, Tumor Necrosis Factor Alpha; IL-1β, Interleukin-1 Beta; TNFRSF1A, Tumor Necrosis Factor Receptor Superfamily Member 1A; TNFRSF1B, Tumor Necrosis Factor Receptor Superfamily Member 1B; MAP3K8, Mitogen-Activated Protein Kinase Kinase Kinase 8; MAP2K7, Mitogen-Activated Protein Kinase Kinase 7.

Out of 119 genes associated with TNF-α-dependent signaling, one-way ANOVA revealed that six genes were significantly differentially expressed (fold change > 5.0 or <–5.0; *p* < 0.05) in G3 and G4 astrocytic tumors relative to G2. *TNF-α* and *IL-1β* were upregulated consistently across both high-grade groups. Additional genes, including *TNFRSF1A*, *TNFRSF1B*, *MAP3K8*, and *MAP2K7*, also demonstrated increased expression (*p* < 0.05). These results suggest progressive activation of inflammatory signaling in higher-grade tumors. Table 1 summarizes the fold changes, while Figure 1 shows RT-qPCR validation.

### 2.2. miRNA-Mediated Regulation of TNF-α Pathway Genes

We next assessed the differential expression of miRNAs predicted to regulate TNF-α signaling components. Downregulation of hsa-miR-34a-3p, a predicted regulator of TNF-α, and members of the miR-30 family targeting *IL-1β*, was observed in G3/G4 compared to G2. Conversely, hsa-miR-106b-5p and hsa-miR-27b-3p, targeting *MAP3K8* and *MAP2K7,* respectively, were upregulated, despite elevated mRNA levels of their targets. No miRNA regulators were predicted for *TNFRSF1A* or *TNFRSF1B*.

These findings are detailed in Table 2.

### 2.3. Methylation Status of TNF-α Pathway Components

To investigate potential epigenetic regulation of inflammatory mediators, we analyzed the promoter methylation status of TNF-α, IL-1β, MAP3K8, and MAP2K7 in astrocytic tumors across different malignancy grades. A clear inverse relationship was observed between promoter methylation and gene expression levels.

In G2 tumors, the majority of cases exhibiting high mRNA expression corresponded to non-methylated promoter regions (15–17 cases per gene), while methylated samples generally showed little or no detectable expression. In G3 tumors, this trend became more variable. IL-1β expression, in particular, was observed in a substantial proportion of methylated cases (*n* = 12), whereas TNF-α, MAP3K8, and MAP2K7 remained primarily expressed in non-methylated samples (11–12 cases). These findings suggest potential gene-specific differences in methylation sensitivity or partial epigenetic silencing in intermediate-grade tumors.

In G4 astrocytomas, a more definitive pattern emerged: expression of all four genes was entirely absent in methylated samples, while all non-methylated cases (*n* = 36) displayed consistent and robust gene expression. This striking dichotomy underscores the functional impact of promoter methylation in advanced tumors and reinforces the notion that epigenetic silencing may contribute to the transcriptional inactivation of key inflammatory mediators in a grade-dependent manner.

**Figure 2:**
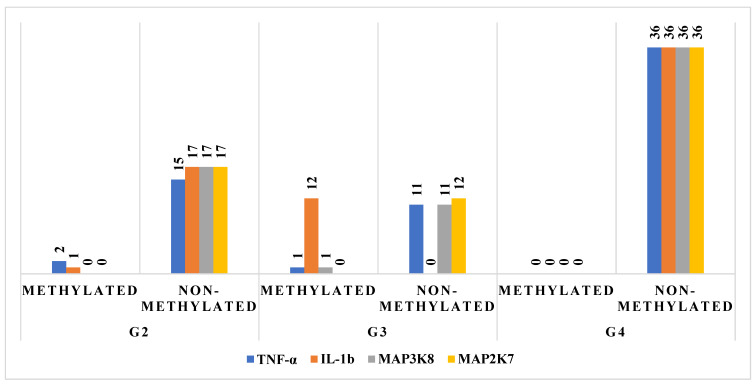
The degree of methylation of selected genes in the G2, G3, and G4 astrocytic tumor samples.

Collectively, these observations support the hypothesis that DNA methylation status plays a significant regulatory role in the expression of TNF-α signaling components during glioma progression. Detailed methylation-expression concordance is illustrated in Figure 2.

### 2.4. Protein Quantification by ELISA

To validate gene-level findings at the protein level, we performed ELISA assays. (Table 3). NF-α and IL-1β levels were significantly increased in G3 and G4 compared to G2 (*p* < 0.05). MAP3K8 protein concentration was similarly elevated, while MAP2K7 showed no significant change. TNFRSF1A and TNFRSF1B trended higher in advanced tumors but did not reach statistical significance.

These results are presented in Table 3.

### 2.5. Protein Expression Validation by Western Blot and Immunohistochemistry

To further validate our transcriptomic findings, we assessed the protein expression levels of TNF-α and IL-1β—two cytokines that showed statistically significant differences in gene expression—using Western blot and immunohistochemistry (IHC) in tumor tissue samples.

**Figure 3:**
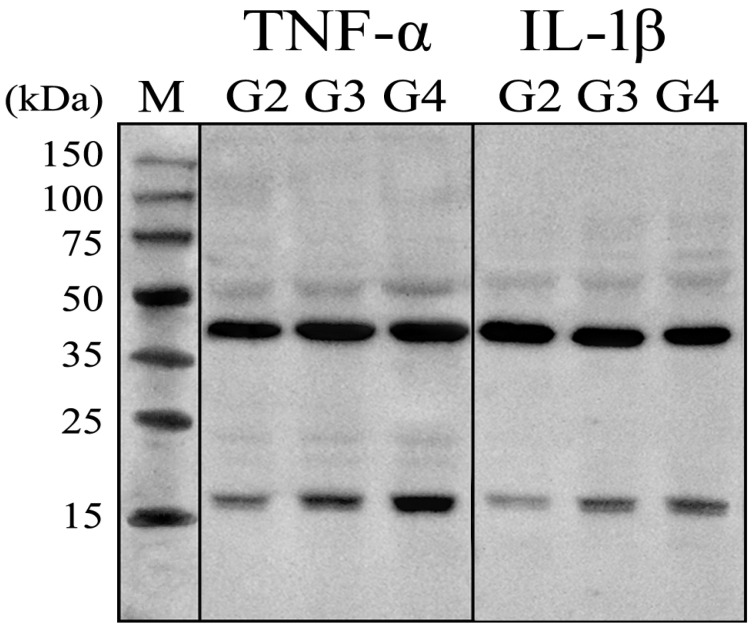
Example of electrophoretic separation of TNF-α and IL-1β in in the G2, G3, and G4 astrocytic tumor samples. TNF-α, Tumor Necrosis Factor Alpha; IL-1β, Interleukin-1 Beta; kDa, kilo Daltons; M, size marker (New England Biolabs Marker).

Figure 3 presents a representative Western blot image, demonstrating the specificity of the antibody-based detection and the integrity of the samples, as evidenced by consistent expression of the housekeeping protein ACTB (β-actin, 42 kDa). Quantitative analysis of the normalized optical density (OD) for TNF-α (17 kDa), relative to ACTB, revealed significantly elevated expression in G3 samples (1.09 ± 0.23), compared to G2 (0.14 ± 0.07, *p* < 0.05) and G4 (1.97 ± 0.54, *p* < 0.05).

Similarly, IL-1β expression was significantly increased in G3 samples (0.48 ± 0.12) compared to both G2 (0.25 ± 0.12, *p* < 0.05) and G4 (0.73 ± 0.18, *p* < 0.05).

**Figure 4:**
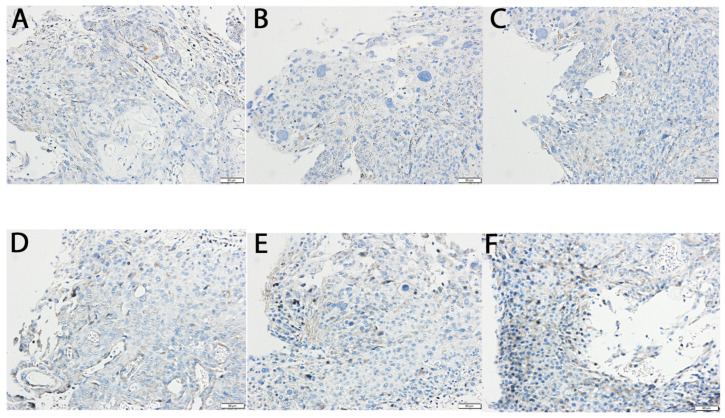
Immunochemical expression of TNF-α and IL-1β in the G2, G3, and G4 astrocytic tumor samples. (**A**) IL-1β expression in a G2 tumor sample; (**B**) IL-1β in G3; (**C**) IL-1β in G4; (**D**) TNF-α expression in G2; (**E**) TNF-α in G3; (**F**) TNF-α in G4. Scale bar = 50 µm. TNF-α, Tumor Necrosis Factor Alpha; IL-1β, Interleukin-1 Beta.

In turn, the optical density of the reaction product for TNF-α in G3 tumor samples reached 128.02 ± 5.67% relative to G2 (*p* < 0.05), while in G4 samples, it increased to 171.43 ± 10.18% of G2 (*p* < 0.05). Similarly, the optical density of the reaction product for IL-1β in G3 samples was 251.79 ± 20.11% compared to G2 (*p* < 0.05), whereas in G4 samples, it further increased to 316.96 ± 21.65% of G2 (*p* < 0.05). Figure 4 illustrates immunohistochemical staining for TNF-α and IL-1β.

### 2.6. Functional Network Analysis of Selected Proteins: Insights from Protein–Protein Interaction (PPI) Analysis

**Figure 5:**
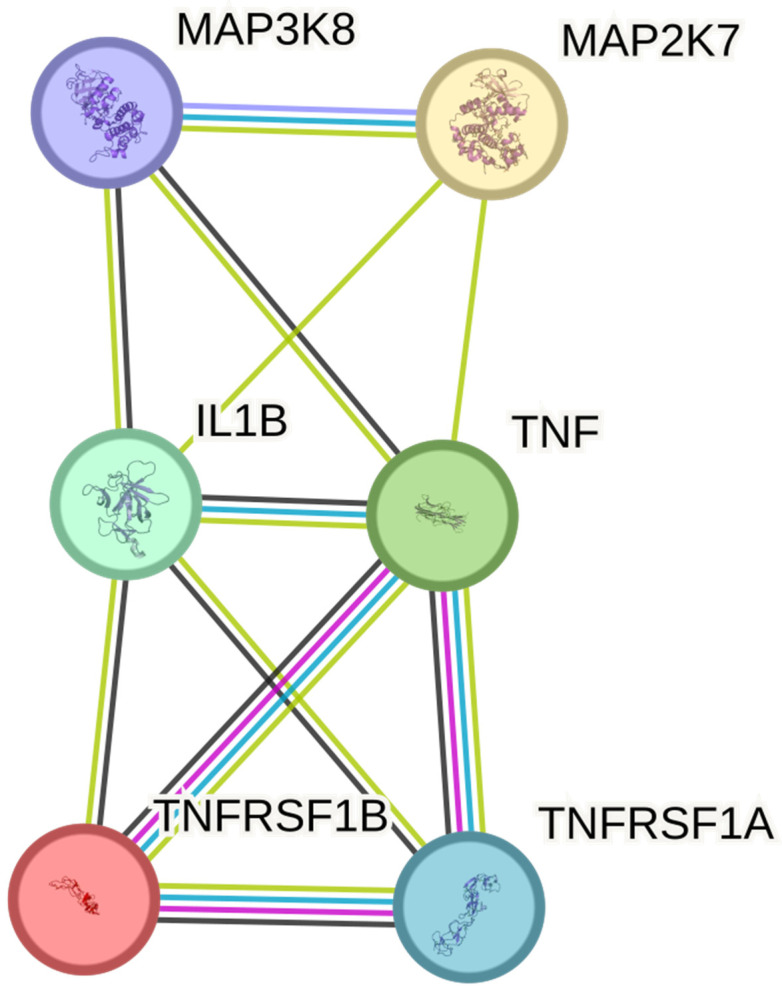
Interaction Network of selected proteins generated using the STRING database. TNF, Tumor Necrosis Factor; IL1B, Interleukin-1 Beta; TNFRSF1A, Tumor Necrosis Factor Receptor Superfamily Member 1A; TNFRSF1B, Tumor Necrosis Factor Receptor Superfamily Member 1B; MAP3K8, Mitogen-Activated Protein Kinase Kinase Kinase 8; MAP2K7, Mitogen-Activated Protein Kinase Kinase 7.

STRING database analysis revealed that the six studied proteins form a tightly connected interaction network (6 nodes, 11 edges; PPI enrichment *p* = 4.48 × 10^−5^), suggesting functional convergence. MAP3K8 was centrally positioned, highlighting its coordinating role in TNF-α-related signaling cascades. Figure 5 shows the STRING-based network.

### 2.7. Kaplan–Meier Survival Analysis and Cox Proportional Hazards Model for TNF-α, IL-1β, TNFRSF1A, TNFRSF1B, MAP3K8, and MAP2K7 in Astrocytic Tumors

**Figure 6:**
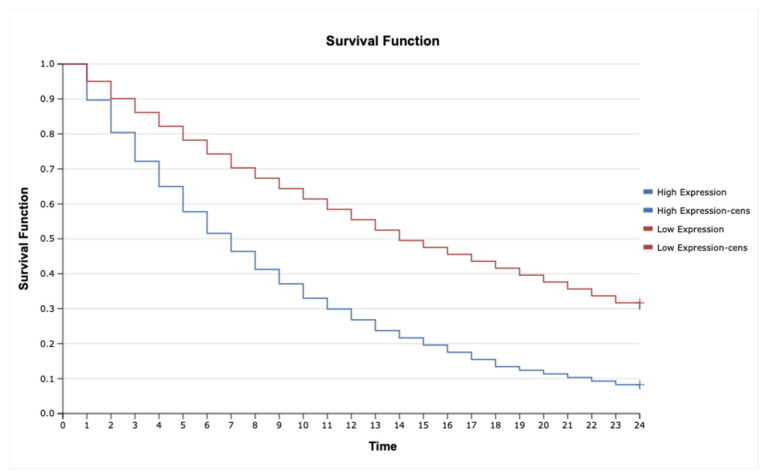
Kaplan–Meier survival curves comparing patients with high and low IL-1β expression.

**Figure 7:**
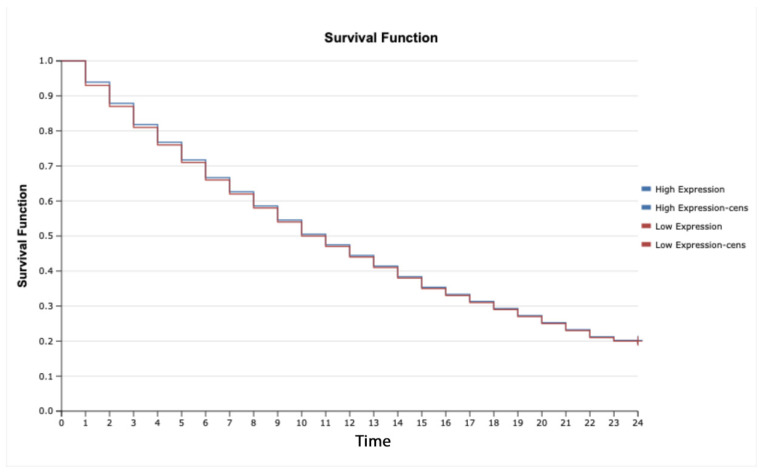
Kaplan–Meier survival curves comparing patients with high and low MAP2K7 expression.

**Figure 8:**
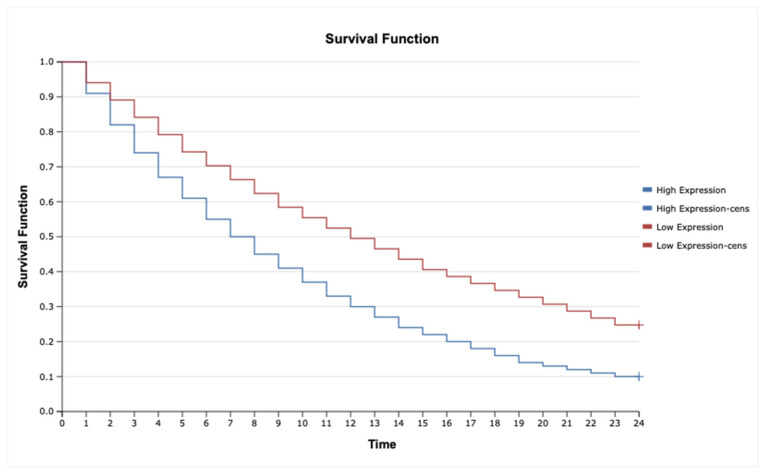
Kaplan–Meier survival curves comparing patients with high and low MAP3K8 expression.

**Figure 9:**
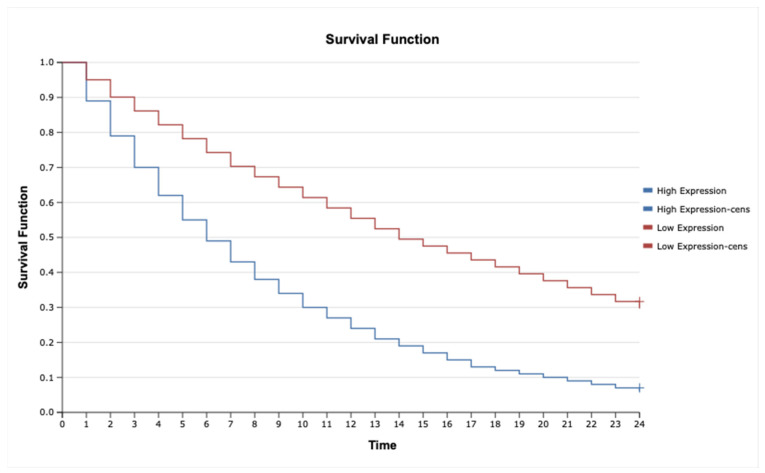
Kaplan–Meier survival curves comparing patients with high and low TNF-α expression.

**Figure 10:**
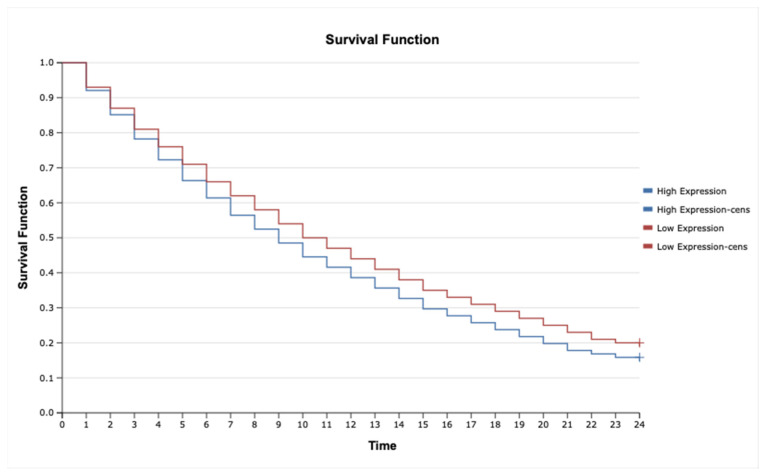
Kaplan–Meier survival curves comparing patients with high and low TNFRSF1A expression.

**Figure 11:**
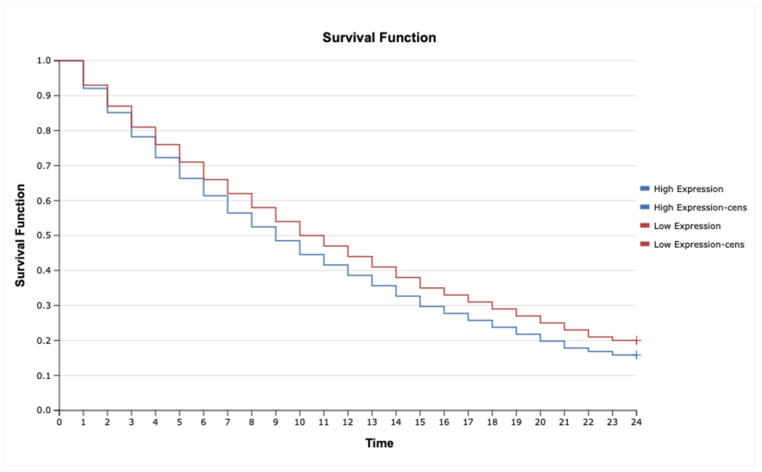
Kaplan–Meier survival curves comparing patients with high and low TNFRSF1B expression.

The analysis revealed that high expression of *IL-1β* (Figure 6; HR = 2.35, 95% CI: 1.45–3.82, *p* = 0.001), *MAP3K8* (Figure 7; HR = 1.88, 95% CI: 1.12–3.16, *p* = 0.015), and *TNF-α* (Figure 8; HR = 2.10, 95% CI: 1.27–3.46, *p* = 0.004) was significantly associated with poorer overall survival. These findings suggest that upregulation of these inflammatory markers may reflect enhanced disease aggressiveness or progression. In contrast, expression levels of *MAP2K7* (Figure 9; HR = 1.05, 95% CI: 0.72–1.52, *p* = 0.36), *TNFRSF1A* (Figure 10; HR = 1.22, 95% CI: 0.81–1.84, *p* = 0.29), and *TNFRSF1B* (Figure 11; HR = 1.18, 95% CI: 0.79–1.76, *p* = 0.33) did not show significant differences in survival between high- and low-expression groups, indicating a limited prognostic utility in this clinical context.

## 3. Discussion

This study provides an integrative, multi-omic evaluation of TNF-α signaling in astrocytic tumors, revealing significant correlations between pathway activation, molecular regulation, and clinical outcome [34,35,36]. Through comprehensive analysis-including RT-qPCR, ELISA, immunohistochemistry, methylation profiling, miRNA expression, and survival analysis, we demonstrate that TNF-α, IL-1 β, and MAP3K8 are not only upregulated in high-grade astrocytomas but also independently predict worse overall survival. These findings reinforce the concept that chronic inflammation, particularly TNF-α-driven, is a key driver of glioma aggressiveness [11,37,38,39].

Chronic inflammation represents a hallmark of GBM, contributing substantially to its pathogenesis and resistance to therapy [40,41]. This proinflammatory state supports immune evasion by GBM cells, fostering immune tolerance and undermining the efficacy of both conventional and targeted treatments [42,43]. The inflammatory microenvironment of GBM is primarily shaped by tumor cells and glioma-associated microglia/macrophages (GAMs), which synergistically promote tumor progression, invasion, and recurrence [44,45]. In addition, GBM cells interact with components of the tumor stroma—including the extracellular matrix, astrocytes, pericytes, and endothelial cells—to secrete a diverse array of cytokines and chemokines. These mediators drive the recruitment and polarization of GAMs [46], creating a self-reinforcing inflammatory loop that exacerbates disease progression and therapeutic resistance.

Our data highlight the TNF-α, IL-1β axis as a critical mediator of glioma progression. Elevated protein and transcript levels of both cytokines in G3 and G4 tumors, coupled with their association with poor survival, are consistent with their established roles in sustaining a tumor-permissive microenvironment [47,48]. TNF-α is a pleiotropic cytokine that, while capable of inducing apoptosis under certain conditions, predominantly fosters tumor survival and immune evasion in the glioma context through NF-kB, MAPK, and JNK signaling [49,50]. IL-1β synergizes with TNF-α to amplify pro-inflammatory gene expression, promote angiogenesis, and remodel the extracellular matrix [51,52,53]. The prognostic value of MAP3K8 (TPL2) further supports this mechanistic axis. MAP3K8 acts as a convergence node for TNF-α and Toll-like receptor (TLR) signaling, triggering ERK and JNK cascades [54,55,56,57], both of which are implicated in glioblastoma stemness, invasion, and resistance to therapy [58,59,60,61]. Notably, while MAP3K8 was significantly elevated and prognostically relevant, MAP2K7, though upregulated, was not associated with survival outcomes-suggesting divergent functional relevance despite shared pathway affiliation [62]. These differences may reflect context-dependent pathway crosstalk, post-translational modifications, or alternative splicing events that modulate kinase function [63,64].

The study further elucidates post-transcriptional regulation via miRNAs. The downregulation of hsa-miR-34a-3p and miR-30e-5p, a known tumor suppressor [65,66,67] targeting TNF-α, aligns with the observed increase in TNF-α expression in higher-grade tumors. Similarly, members of the miR-30 family, particularly miR-30e-3p, which are predicted to target IL-1β, were markedly suppressed in G3/G4 tumors. These findings support a model wherein miRNA silencing of cytokines is progressively lost during malignant transformation, contributing to unchecked inflammatory signaling [68,69].

Conversely, miR-106b-5p and miR-27b-3p, predicted to regulate MAP3K8 and MAP2K7, were paradoxically upregulated despite increased mRNA and protein levels of their targets. This suggests non-canonical regulation or the presence of miRNA sponging mechanisms, such as competing endogenous RNAs (ceRNAs), circular RNAs, or long non-coding RNAs that sequester miRNAs [70,71,72]. These mechanisms remain underexplored in gliomas and represent a potential gap in our understanding of miRNA-mediated regulation.

The epigenetic data add a further layer of complexity. Our MS PCR analysis showed that promoter methylation suppressed gene expression across the examined cytokines and kinases, particularly in lower-grade tumors. In contrast, high-grade gliomas exhibited hypomethylation and strong gene expression, consistent with the broader observation of global hypomethylation and locus-specific hypermethylation in malignant gliomas. Interestingly, IL-1β maintained expression even in methylated G3 samples, indicating potential methylation-independent activation, possibly via enhancer remodeling or inflammatory stimulus-induced transcription [73,74,75,76].

The identification of TNF-α and MAP3K8 as independent predictors of poor prognosis has significant translational potential. Targeting TNF-α signaling in gliomas has been challenging due to its dual role in tumor biology and host immunity [50,77,78]. However, selective inhibitors of MAP3K8, which acts downstream of multiple inflammatory inputs, may provide a more precise intervention point. Importantly, MAP3K8 inhibitors have shown promise in preclinical models of inflammatory diseases and hematologic malignancies, but their application in gliomas remains untested [79,80,81]. Our results suggest that a subset of patients with high MAP3K8 expression could benefit from such approaches [62].

Moreover, integrating miRNA and methylation profiles with standard diagnostic workflows may enhance the molecular stratification of astrocytic tumors, allowing for more personalized prognostication and treatment. For example, downregulation of miR-34a or miR-30e-3p could be explored as biomarkers for cytokine-driven gliomas, while methylation signatures may guide epigenetic therapy approaches [82,83,84].

Despite these strengths, several limitations merit discussion. First, while our study included a well-characterized cohort, the sample size was moderate, potentially limiting statistical power—particularly for subgroup analyses and survival modeling. Second, although we observed robust correlations between gene expression and clinical outcomes, the relationships remain inherently associative. Causal links, particularly between MAP3K8, miRNA regulation, and glioma progression, require functional validation in cell-based and in vivo models. Approaches such as CRISPR/Cas9-mediated gene knockouts, cytokine inhibition assays, or promoter-reporter luciferase assays would greatly strengthen mechanistic interpretations. Third, the methylation analysis was limited to selected CpG regions; genome-wide methylation profiling could uncover additional regulatory elements or enhancer interactions affecting transcriptional activity.

Additionally, the use of bulk tumor tissue represents an important constraint, as it prevents discrimination between tumor-intrinsic gene expression and signals derived from non-neoplastic cells in the microenvironment. Given the centrality of inflammation in gliomagenesis, distinguishing the contributions of glioma cells from those of infiltrating immune cells (e.g., microglia, macrophages) is particularly critical. Future studies employing single-cell RNA sequencing or spatial transcriptomic technologies will be essential for resolving cell-type–specific signaling networks and dissecting the inflammatory ecosystem of astrocytic tumors.

Finally, it should be noted that the use of glyceraldehyde-3-phosphate dehydrogenase (GAPDH) and beta actin (*ACTB*) as internal controls for Western blot normalization, while conventional and widely accepted, is not without limitations. Both of these housekeeping proteins have been reported to exhibit variable expression in certain pathological conditions, including a range of cancers. Specifically, studies have demonstrated that GAPDH and β-actin levels can be upregulated or downregulated depending on tumor type, grade, metabolic status, and cellular stress responses [85,86]. In the context of astrocytic tumors, where metabolic reprogramming and cytoskeletal remodeling are frequently observed, such fluctuations in housekeeping protein levels could potentially introduce bias in quantitative protein analyses. Despite these concerns, GAPDH and β-actin remain widely used due to their robust detection, historical precedent, and relative expression stability across many sample types. However, we acknowledge this limitation in our study and suggest that future investigations should consider validating the stability of internal reference proteins in the specific tissue and disease context under study.

A further gap lies in the spatial and cellular resolution of cytokine expression. Bulk tissue analysis does not distinguish between tumor cell-derived and microenvironmental cytokine sources (e.g., microglia, macrophages). Future studies employing single-cell RNA-seq or spatial transcriptomics could clarify the cellular origins of TNF-α, IL-1β in gliomas and their interaction with infiltrating immune cells.

## 4. Materials and Methods

### 4.1. Characterization of Participants

This study included tumor samples from 60 patients who underwent surgical resection of astrocytic brain tumors at two neurosurgical departments in Kraków, Poland: the Department of Neurosurgery at the 5th Military Clinical Hospital with the SP ZOZ Polyclinic and the Department of Neurosurgery at Saint Raphael Hospital.

Preoperative diagnosis was initially established through contrast-enhanced computed tomography (CT) and confirmed with magnetic resonance imaging (MRI). Imaging protocols incorporated T1- and T2-weighted sequences, fluid-attenuated inversion recovery (FLAIR), and, when clinically indicated, diffusion tensor imaging. For tumors adjacent to functionally critical brain regions, additional evaluations such as functional MRI and tractography supported surgical planning via neuronavigation.

Surgical intervention prioritized maximal tumor resection with preservation of neurological function. Intraoperative guidance included frameless neuronavigation, fluorescence-guided resection with 5-aminolevulinic acid (5-ALA) in high-grade gliomas, and cortical mapping through direct electrical stimulation when operating near sensorimotor areas.

Postoperative tumor classification was based on histopathological assessment according to the World Health Organization (WHO) grading system, categorizing tumors into grade II (G2), grade III (G3), and grade IV (G4) astrocytomas.

The study cohort comprised 35 female and 30 male patients, aged between 56 and 59 years on average. Among females, tumor distribution was: 10 G2, 7 G3, and 18 G4. For males: 7 G2, 5 G3, and 18 G4 cases were identified.

Patient eligibility was determined using strict inclusion and exclusion criteria. Inclusion required scheduled elective tumor resection at one of the participating centers and provision of informed consent. Patients with concurrent malignancies were excluded from the study.

To ensure consistency in perioperative conditions, all patients adhered to a standardized fasting protocol, with the final preoperative meal administered by 6:00 PM on the evening before surgery. All procedures were conducted between 8:00 A.M. and 11:00 A.M. the following day under elective surgical scheduling.

### 4.2. Extraction of Total Ribonucleic Acid (RNA)

Tumor tissue samples were homogenized using a T18 Digital Ultra-Turrax rotor–stator system (IKA Poland Ltd., Warsaw, Poland). Total RNA was extracted using TRIzol reagent (Invitrogen, Carlsbad, CA, USA), followed by further purification with the RNeasy Mini Kit (QIAGEN, Hilden, Germany) to ensure removal of proteins and phenolic contaminants. Residual genomic DNA was eliminated through DNase I treatment (Fermentas, Burlington, ON, Canada).

RNA quality and integrity were assessed via 1% agarose gel electrophoresis stained with ethidium bromide (0.5 µg/mL). RNA concentrations were determined by measuring absorbance at 260 nm using a spectrophotometer, ensuring consistent purity for downstream applications.

### 4.3. Microarray Analysis

Gene expression profiling was conducted to compare transcript levels between astrocytic tumor samples and non-tumor control tissues. The Affymetrix HG-U133A2 platform and GeneChip™ 3′ IVT PLUS Reagent Kit (Affymetrix, Santa Clara, CA, USA; Cat. No. 902416) were used in accordance with the manufacturer’s protocols and previously validated procedures [87]. The Kyoto Encyclopedia of Genes and Genomes (KEGG) pathway database [88] was used to identify genes involved in the TNF-α signaling cascade. Specifically, the KEGG TNF-α signaling pathway map (entry hsa04668, pathway ID N00056) served as the reference for compiling a curated list of 119 genes functionally associated with this pathway.

### 4.4. Microarray Profiling of TNF-α-Related miRNAs

MicroRNA expression analysis was performed using the GeneChip miRNA 2.0 Array (Affymetrix), enabling comprehensive detection of differentially expressed miRNAs between tumor and control tissues. The experimental workflow followed the manufacturer’s standardized protocol to ensure data reliability and reproducibility. miRNA–mRNA interaction predictions were carried out using the TargetScan (http://www.targetscan.org/, accessed on 10 March 2025) [89] and miRanda (http://mirdb.org, accessed on 10 March 2025)—[90] databases. High-confidence target interactions were defined by a prediction score > 80, while interactions with scores < 60 were flagged for additional validation [90,91].

### 4.5. Validation of Gene Expression Using Quantitative Reverse-Transcription Polymerase Chain Reaction (qRT-PCR)

To validate the microarray data, qRT-PCR was performed on selected genes using the SensiFast SYBR Quantitative reverse transcription PCR (qRT-PCR) to validate selected gene expression profiles from the microarray analysis. Reactions were performed using the SensiFast SYBR No-ROX One-Step Kit (Bioline, London, UK). The thermal protocol included reverse transcription at 45 °C, polymerase activation at 95 °C for 2 min, followed by 40 amplification cycles: 95 °C for 5 s, 60 °C for 10 s, and 72 °C for 5 s.

Expression levels were normalized to GAPDH *ACTB* as an internal control. Relative quantification was calculated using the 2^−ΔΔCt^ method. A fold-change threshold of 1 represented baseline expression; values above or below indicated overexpression or downregulation, respectively. Primer sequences are listed in Table 4.

### 4.6. Methylation Analysis of Genes

CpG-rich regions within the target genes were identified using the MethPrimer tool (http://www.urogene.org/cgi-bin/methprimer/methprimer.cgi; accessed on 19 January 2025) [92]. Primer design criteria included a CpG island length > 100 bp, GC content > 50%, and an observed-to-expected CpG ratio > 0.6 (see Table 5).

Genomic DNA was subjected to sodium bisulfite conversion followed by cleanup, as per the kit instructions. Methylation-specific PCR (MSP) was performed using the QuantiTect SYBR Green PCR Kit (QIAGEN, Hilden, Germany). Thermal cycling consisted of an initial 5 min denaturation at 95 °C, followed by 40 cycles at 94 °C (30 s), 65 °C (30 s), and 72 °C (30 s).

PCR products were analyzed by agarose gel electrophoresis (1% gel, ethidium bromide 0.5 µg/mL, 1× TBE buffer, 120 V). Fragment sizes were compared to the pBR322/HaeIII marker. To validate amplification specificity, methylated and non-methylated DNA controls from the EpiTect Control DNA Set (QIAGEN) were included.

### 4.7. Enzyme-Linked Immunosorbent Assay (ELISA) Quantification of Selected Proteins in G2, G3, G4 Astrocytic Tumor Samples

Quantitative analysis of selected inflammatory and signaling proteins in astrocytic tumor tissues (grades G2, G3, and G4) was performed using commercially available ELISA kits, following the manufacturers’ protocols. The concentrations of TNF-α and IL-1β were determined using the Human TNF alpha ELISA Kit (Invitrogen Life Technologies, Carlsbad, CA, USA; catalog number: KHC3012) and the Human IL-1 beta ELISA Kit (Invitrogen Life Technologies, Carlsbad, CA, USA; catalog number: BMS224-2), respectively. To assess the involvement of TNF receptors in astrocytic tumor progression, levels of tumor necrosis factor receptor superfamily member 1A (TNFRSF1A) and member 1B (TNFRSF1B) were measured using specific ELISA kits obtained from Invitrogen (catalog numbers: BMS224-2 and KAC1771, respectively). Additionally, components of the MAPK signaling pathway were evaluated. Human MAP2K7 concentrations were determined using the Human MAP2K7 ELISA Kit (MyBioSource, San Diego, CA, USA; catalog number: MBS9334956), while Human MAP3K8 levels were quantified with the Human MAP3K8 ELISA Kit (MyBioSource, San Diego, CA, USA; catalog number: MBS9325582). Each assay was conducted in triplicate depending on sample availability, and absorbance was measured at the recommended wavelength using a microplate reader. Protein concentrations were calculated based on standard curves generated with known concentrations of the target analytes.

### 4.8. Western Blot for Protein Quantification of TNF-α and IL-1β

TNF-α was detected using a specific antibody (STI, Poznań, Poland; bs-10802R) at a 1:1000 dilution, while IL-1β was assessed using a primary antibody from Labiot (Cieszyn, Poland; Cell Signaling; d3ue2) also at a 1:1000 dilution. Protein separation was performed by SDS-PAGE using polyacrylamide gels.

Glyceraldehyde 3-phosphate dehydrogenase (GAPDH; sc-47724, Santa Cruz Biotechnology, Dallas, TX, USA; 1:500 dilution) served as the endogenous control. For Western blot detection, a horseradish peroxidase (HRP)-conjugated goat anti-rabbit IgG secondary antibody (BioRad, Milan, Italy; catalog no. 1706515; 1:3000 dilution) was used. Absorbance readings for ELISA were obtained at 540 nm using an M200PRO microplate reader (Tecan, Männedorf, Switzerland). Detailed ELISA and Western blot procedures were conducted according to protocols described in previously published studies.

### 4.9. Immunohistochemical (IHC) Analysis of TNF-α and IL1β in Astrocytic Brain Tumor Samples

Paraffin-embedded tissue specimens were sectioned into 8.0 µm thick slices using a rotary microtome (Leica Microsystems, Germany). Deparaffinization, antigen retrieval, and antibody incubation steps were performed following the manufacturer’s protocols provided with the DAB Substrate Kit (Peroxidase, HRP; Vector Laboratories, Newark, CA, USA) and the IHC-Paraffin Protocol (Abcam plc, Cambridge, UK).

Immunoreactive signals were visualized and documented using a Nikon Coolpix-equipped fluorescent microscope. Cellular localization(Melville, NY 11747-3064, USA) and protein expression levels were analyzed using computer-assisted image analysis in ImageJ software (version: 1.x). Representative images were acquired from three non-overlapping fields at 200× magnification.

Quantification of the DAB chromogen signal was performed using the IHC-Profiler plug-in in ImageJ. Optical density was measured in regions exhibiting immunoreactivity, and the average percentage of DAB-stained area was calculated in relation to the background staining intensity for each image field.

### 4.10. Functional Network Analysis of Protein–Protein Interactions (PPI)

To assess the functional relationships among the selected proteins (TNF-α, IL-1β, TNFRSF1A, TNFRSF1B, MAP3K8, and MAP2K7), we used the STRING database (Search Tool for the Retrieval of Interacting Genes/Proteins, version 12.0; https://string-db.org, accessed on 10 March 2025) [93]. The official gene symbols of the six proteins were entered into the STRING search tool, and interactions were explored based on known and predicted associations, including physical binding and functional co-expression.

The analysis was performed using the full STRING network with a medium confidence score threshold (≥0.4). Edges in the resulting network were displayed according to the type of supporting evidence, allowing visualization of both direct (physical) and indirect (functional) associations. Topological features of the network, including the number of nodes, number of edges, average node degree, and local clustering coefficient, were automatically calculated. The statistical significance of the interaction enrichment was assessed using the STRING-provided PPI enrichment *p*-value, which determines whether the observed number of connections exceeds what would be expected for a randomly selected set of proteins of similar size.

### 4.11. Statistical Analysis

All statistical analyses were performed using StatPlus (AnalystSoft Inc., Brandon, FL, USA, https://www.analystsoft.com/en/products/statplusmacle/, accessed on 10 March 2025) and the Transcriptome Analysis Console (Affymetrix, Santa Clara, CA, USA). Prior to analysis, data distributions were assessed for normality using the Shapiro–Wilk test. A threshold of *p* < 0.05 was used to indicate non-normal distribution.

For comparisons of gene and protein expression levels among tumor grades (G2, G3, G4), one-way analysis of variance (ANOVA) was applied. To control for false discovery rate due to multiple testing, the Benjamini–Hochberg correction was employed. Scheffé’s post hoc test was used to identify specific between-group differences.

Kaplan–Meier survival analysis was performed to evaluate associations between molecular markers and overall survival. Differences in survival curves were compared using the log-rank test. Furthermore, Cox proportional hazards regression models were used to estimate hazard ratios (HRs) and 95% confidence intervals (CIs), allowing multivariate assessment of the prognostic significance of cytokine expression levels, miRNA profiles, and methylation status.

All results were considered statistically significant at *p* < 0.05. Data are presented as mean ± standard deviation (SD) unless otherwise stated.

## 5. Conclusions

In summary, our study demonstrates that dysregulation of the TNF-α signaling axis—including upregulation of TNF-α, IL-1β, and MAP3K8—is a hallmark of high-grade astrocytomas and correlates with worse patient outcomes. This dysregulation is shaped by miRNA suppression and epigenetic modifications, emphasizing the multilayered regulation of inflammatory signaling in glioma. These findings highlight promising targets for molecular therapy and lay the groundwork for further mechanistic exploration in translational glioma research.

## Figures and Tables

**Table 1:** Expression profile of genes significantly differentiating G3/G4 vs. G2 samples based on microarray analysis.

ID	mRNA	log_2_FC G3 vs. G2	log_2_FC G4 vs. G2
207113_s_at	*TNF-α*	5.98 ± 0.34	8.19 ± 0.33
1563357_at		5.89 ± 0.43	8.76 ± 0.13
39402_at	*IL-1β*	5.56 ± 0.56	7.23 ± 0.98
207643_s_at	*TNFRSF1A*	5.09 ± 0.71	5.56 ± 0.77
203508_at	*TNFRSF1B*	5.10 ± 0.81	5.51 ± 0.98
205027_s_at	*MAP3K8*	5.11 ± 0.44	5.87 ± 0.19
235421_at		5.14 ± 0.67	5.67 ± 0.34
209951_s_at	*MAP2K7*	5.33 ± 0.81	5.71 ± 0.55
209952_s_at		5.43 ± 0.19	6.03 ± 0.63
216206_x_at		5.16 ± 0.34	6.01 ± 0.76
226023_at		5.77 ± 0.81	5.61 ± 0.99
226053_at		5.18 ± 0.18	5.78 ± 1.01

TNF-α, Tumor Necrosis Factor Alpha; IL-1β, Interleukin-1 Beta; TNFRSF1A, Tumor Necrosis Factor Receptor Superfamily Member 1A; TNFRSF1B, Tumor Necrosis Factor Receptor Superfamily Member 1B; MAP3K8, Mitogen-Activated Protein Kinase Kinase Kinase 8; MAP2K7, Mitogen-Activated Protein Kinase Kinase 7.

**Table 2:** Expression of miRNAs potentially involved in the regulation of the studied genes.

mRNA	*miRNA*	Target Score	G3 vs. G2 (FC)	G4 vs. G2 (FC)
*TNF-α*	hsa-miR-34a-3p	96	−2.42 ± 0.71	−2.12 ± 0.34
*IL-1β*	hsa-miR-30a-3p	83	−1.72 ± 0.21	−2.87 ± 0.21
	hsa-miR-30d-3p		−1.88 ± 0.19	−2.10 ± 0.19
	hsa-miR-30e-3p		−3.45 ± 0.33	−3.78 ± 0.78
*MAP3K8*	hsa-miR-106b-5p	86	+1.98 ± 0.18	+2.01 ± 0.42
*MAP2K7*	hsa-miR-27b-3p	86	+1.71 ± 0.51	+2.43 ± 0.10

TNF-α, Tumor Necrosis Factor Alpha; IL-1β, Interleukin-1 Beta; MAP3K8, Mitogen-Activated Protein Kinase Kinase Kinase 8; MAP2K7, Mitogen-Activated Protein Kinase Kinase 7.

**Table 3:** Concentration of TNF-α, IL-1β, TNFRSF1A, TNFRSF1B, MAP3K8, and MAP2K7 protein in astrocytic tumor samples.

Protein	G2	G3	G4
TNF-α [pg/mL]	237.89 ± 19.12	376.12 ± 21.87 *	381.91 ± 23.98 *
IL-1β [pg/mL]	67.12 ± 8.19	89.91 ± 10.41 *	100.12 ± 12.56 *
TNFRSF1A [ng/mL]	1.23 ± 0.11	1.45 ± 0.09	1.76 ± 0.23
TNFRSF1B [ng/mL]	0.98 ± 0.12	1.34 ± 0.22	1.50 ± 0.19
MAP3K8 [ng/mL]	13.98 ± 0.98	18.71 ± 1.23	19.18 ± 1.34
MAP2K7 [ng/mL]	17.18 ± 1.78	18.98 ± 2.71	18.45 ± 2.09

TNF-α, Tumor Necrosis Factor Alpha; IL-1β, Interleukin-1 Beta; TNFRSF1A, Tumor Necrosis Factor Receptor Superfamily Member 1A; TNFRSF1B, Tumor Necrosis Factor Receptor Superfamily Member 1B; MAP3K8, Mitogen-Activated Protein Kinase Kinase Kinase 8; MAP2K7, Mitogen-Activated Protein Kinase Kinase 7; * *p* < 0.05 vs. G2.

**Table 4:** Nucleotide sequence of primers used in RTqPCR.

mRNA	RT-qPCR Amplification Primers (5′-3′)
*TNF-α*	Forward: AGGACCAGCTAAGAGGGAGA Reverse: CCCGGATCATGCTTTCAGTG
*IL-1β*	Forward: GGAGAATGACCTGAGCACCT Reverse: GGAGGTGGAGAGCTTTCAGT
*TNFRSF1A*	Forward: GCTGTACCAAGTGCCACAAA Reverse: CTCCACCTGACCCATTTCCT
*TNFRSF1B*	Forward: TTGTGTCTGCGTCTGTGTTG Reverse: GCTACACTGGTTTCCCCTCT
*MAP3K8*	Forward: GGCCATTCAACCAAAGCAGA Reverse: GGTTTCTCTCCAGGGAAGCT
*MAP2K7*	Forward: GGGACGTTCATCACCAACAC Reverse: CTTCAGGTAGTACAGCGCCT
*ACTB*	Forward: TCACCCACACTGTGCCCATCTACGA Reverse: CAGCGGAACCGCTCATTGCCAATGG
*GAPDH*	Forward: GGCCAGATCCTGTCCAAGC Reverse: GTGGGTTTCCACCATTAGCAC

TNF-α, Tumor Necrosis Factor Alpha; IL-1β, Interleukin-1 Beta; TNFRSF1A, Tumor Necrosis Factor Receptor Superfamily Member 1A; TNFRSF1B, Tumor Necrosis Factor Receptor Superfamily Member 1B; MAP3K8, Mitogen-Activated Protein Kinase Kinase Kinase 8; MAP2K7, Mitogen-Activated Protein Kinase Kinase 7; ACTB, beta actin; GAPDH, glyceraldehyde-3-phosphate dehydrogenase.

**Table 5:** Characteristics of primers designed for the MSP.

mRNA	M/U	NCBI Reference Sequen	Primers (5′-3′)
*TNF-α*	M	NM_000594.4	Forward: GAGTATTGAAAGTATGATTCGGGAC Reverse: CAAAATACAACAAACAAAAAAACGTA
	U		Forward: GTATTGAAAGTATGATTTGGGATGT Reverse: AAAATACAACAAACAAAAAAACATA
*IL-1β*	M	NM_000576	Forward: ATAGTAAGGGTTTTAGGTAGGTCGC Reverse: CGAAACGTACAATTCAATAATCGTA
	U		Forward: AGTAAGGGTTTTAGGTAGGTTGTGT Reverse: CCAAAACATACAATTCAATAATCATA
*TNFRSF1A*	M	NM_001065.4	Forward: GAGGCGTAATATAGTATGTTGGC Reverse: TCATCTAAAAAAACTAAACGCGAA
	U		Forward: GAGGTGTAATATAGTATGTTGGTGA Reverse: ACCTCATCTAAAAAAACTAAACACAAA
*TNFRSF1B*	M	NM_001066.3	Forward: GTTGAGTAAGTAGGAGGGGTGTC Reverse: ACTCCGAATAAAAAACGTAAACGTA
	U		Forward: GTTGAGTAAGTAGGAGGGGTGTT Reverse: ACTCCAAATAAAAAACATAAACATA
*MAP3K8*	M	NM_005204.4	Forward: TCGTCGGATTTTAGTGGTTC Reverse: AAAAATTACATCTACGACCTTAACG
	U		Forward: TGTTGGATTTTAGTGGTTTG Reverse: AAATTACATCTACAACCTTAACACT
*MAP2K7*	M	NM_001297555.2	Forward: GTATTTTGGGTAAGATGATAGTGGC Reverse: CTTAACTTTAAAATCCACCAAACGA
	U		Forward: TATTTTGGGTAAGATGATAGTGGTG Reverse: TTAACTTTAAAATCCACCAAACAAC

M, primers designed for methylated sequences; U, primers designed for non-methylated sequence; TNF-α, Tumor Necrosis Factor Alpha; IL-1β, Interleukin-1 Beta; TNFRSF1A, Tumor Necrosis Factor Receptor Superfamily Member 1A; TNFRSF1B, Tumor Necrosis Factor Receptor Superfamily Member 1B; MAP3K8, Mitogen-Activated Protein Kinase Kinase Kinase 8; MAP2K7, Mitogen-Activated Protein Kinase Kinase 7.

## Data Availability

The data used to support the findings of this study are included in the article. The data cannot be shared due to third-party rights and commercial confidentiality.

## Abbreviations

The following abbreviations are used in this manuscript:

TNF-α	Tumor Necrosis Factor Alpha
IL-1β	Interleukin-1 Beta
TNFRSF1A	Tumor Necrosis Factor Receptor Superfamily Member 1A
TNFRSF1B	Tumor Necrosis Factor Receptor Superfamily Member 1B
MAP3K8	Mitogen-Activated Protein Kinase Kinase Kinase 8
MAP2K7	Mitogen-Activated Protein Kinase Kinase 7
NF-κB	Nuclear Factor kappa-light-chain-enhancer of activated B cells
JNK	c-Jun N-terminal Kinase
RT-qPCR	Reverse Transcription Quantitative Polymerase Chain Reaction
ELISA	Enzyme-Linked Immunosorbent Assay
IHC	Immunohistochemistry
MSP	Methylation-Specific Polymerase Chain Reaction
PPI	Protein–Protein Interaction
KEGG	Kyoto Encyclopedia of Genes and Genomes
GAPDH	Glyceraldehyde-3-Phosphate Dehydrogenase
ACTB	Beta Actin
miRNA	MicroRNA
ceRNA	Competing Endogenous RNA
IDH	Isocitrate Dehydrogenase
MGMT	O6-Methylguanine-DNA Methyltransferase
EGFR	Epidermal Growth Factor Receptor
GBM	Glioblastoma Multiforme
G2	Grade II Astrocytoma
G3	Grade III Astrocytoma
G4	Grade IV Astrocytoma
HR	Hazard Ratio
CI	Confidence Interval
SD	Standard Deviation
SP ZOZ	Samodzielny Publiczny Zakład Opieki Zdrowotnej
